# Enhancing the photovoltaic performance of hybrid heterojunction solar cells by passivation of silicon surface via a simple 1-min annealing process

**DOI:** 10.1038/s41598-019-48504-7

**Published:** 2019-08-19

**Authors:** Rongbin Xie, Naoya Ishijima, Hisashi Sugime, Suguru Noda

**Affiliations:** 1 Department of Applied Chemistry, Waseda University, Tokyo, 169-8555 Japan; 2 Waseda Research Institute for Science and Technology, Waseda University, Tokyo, 169-8555 Japan

**Keywords:** Solar cells, Solar cells

## Abstract

Solution-based heterojunction technology is emerging for facile fabrication of silicon (Si)-based solar cells. Surface passivation of Si substrate has been well established to improve the photovoltaic (PV) performance for the conventional bulk Si cells. However, the impact is still not seen for the heterojunction cells. Here, we developed a facile and repeatable method to passivate the Si surface by a simple 1-min annealing process in vacuum, and integrated it into the heterojunction cells with poly(3,4-ethylenedioxythiophene): poly(styrenesulfonate) (PEDOT:PSS) or carbon nanotube (CNT). A thin and dense oxide layer was introduced on the Si surface to provide a high-quality hole transport layer and passivation layer. The layer enhanced the power conversion efficiency from 9.34% to 12.87% (1.38-times enhancement) for the PEDOT:PSS/n-Si cells and from 6.61% to 8.52% (1.29-times enhancement) for the CNT/n-Si cells. The simple passivation is a promising way to enhance the PV performance of the Si cells with various solution-based heterojunctions.

## Introduction

Solar energy is one of the sustainable and renewable energy sources for generating electricity and clean fuels. To meet the increasing demand for energy, researchers have paid enormous attention to the solar cells which can harvest sun’s inexhaustible energy and convert it into electricity by photovoltaic (PV) effect^1^. Nowadays, silicon (Si) remains the most widely employed material in solar cells manufacturing due to the low cost, lack of toxicity, and small electronic band gap from near-infrared to visible light^2,3^. However, one of the main limitations of Si solar cells is their high temperature and complex manufacturing process^4^. Therefore, simple solution-based fabrication of Si heterojunction solar cells is a promising method to further reduce the cost of the PV devices.

Heterojunction solar cells enable high power conversion efficiency (PCE) by making charge carrier selective contacts to the absorber materials^5^. Recently, Si solar cells hybridized with organic semiconductors^6–8^, quantum dots^9–11^, and carbon nanomaterials^12–14^ have attracted a great deal of research interest owing to their promise of room-temperature manufacturing and solution processing capability. As one of the new type low-cost solar cells, the poly(3,4-ethylenedioxythiophene): poly(styrenesulfonate) (PEDOT:PSS) based Si solar cell showed an encouraging PCE of above 14%, significantly promoting the development of Si heterojunction solar cells^15^. Previously, we demonstrated solution-processed carbon nanotube (CNT)/n-Si heterojunction solar cells with a PCE of 10.4% based on the textured n-type Si wafer and p-type CNT films^16^. Surface passivation of Si substrate has been established well for the PV performance enhancement of the conventional bulk Si cells but was usually overlooked in these heterojunction cells. Pristine Si usually has high concentration non-saturated dangling bonds at the surface that cause high local carrier recombination rates^17,18^. Inserting a thin passivation layer between Si and contact materials could provide an intermediate “i” region for the p-i-n device, which greatly suppresses the carrier recombination and builds an internal electrical field^19–21^. For conventional bulk Si solar cells, various approaches such as thermal oxidation, plasma enhanced chemical vapor deposition, atomic layer deposition have been shown to be effective in performance enhancement, but such passivation methods are based on long time, high temperature and/or complex processes^2,22^. To realize the new solution-based heterojunction solar cells, surface passivation has been studied by exposure to the ambient atmosphere or chemical oxidation. However, these methods are very sensitive to the environmental conditions, leading to a low-quality passivation layer^23,24^. Therefore, it is essential to realize a high-quality passivation layer via a simple process.

Herein, we passivated the n-Si substrate surface with a thin and dense oxide layer by a quick annealing process in vacuum (1 min, 500–550 °C, <5 × 10^−4^ Pa), which will greatly reduce the passivation time and cost. Moreover, we integrated the surface-passivated n-Si with two types of heterojunctions: PEDOT:PSS/n-Si and CNT/n-Si. The simple passivation proved effective in enhancing the fill factor (FF) and PCE. Future prospect for the low-cost, flexible thin-film Si solar cells is also discussed.

## Methods

### Passivation of n-Si substrate

n-Si (100) substrate (1–5 Ω cm with P as dopant) that has a 500 nm-thick thermal oxide layer (SiO_2_) with an opening (ϕ = 2 mm, active area of 3.14 mm^2^) was used. It was dipped into 4.7 wt% hydrofluoric acid (HF) for 30 s and rinsed in isopropanol (IPA) and purified water to remove the native oxide layer on the opening. Then the n-Si substrate was set in a vacuum chamber, whose base pressure was kept at 5 × 10^−4^ Pa by a turbo molecular pump. Annealing was performed by heating the n-Si substrate at ~400 °C/min to a target temperature *T* of 400–800 °C, holding it at *T* for a target time *t* of 0–10 min, and cooled down at ~400 °C/min below 400 °C (Supplementary Information, Fig. S1). During the annealing process, the pressure increased with temperature to 2 × 10^−2^ Pa at maximum.

### Fabrication of PEDOT:PSS/n-Si solar cells

PEDOT:PSS aqueous dispersion (200 μL, 500 S/cm, Heraeus Deutschland GmbH & Co. KG, Leverkusen, Germany) was mixed with IPA (50 vol%). The mixture was spin-coated onto the passivated n-Si substrate at 2000 rpm for 60 s, and subsequently annealed on a hot plate at 130 °C for 10 min in ambient air. To reduce the edge effect and avoid the short circuit, we covered the edges of the n-Si substrate by a mask during the spin-coating process. To complete the solar cell, a top gold (Au) anode with a 2.2 mm-sized opening (square or circle) was formed onto the PEDOT:PSS by sputtering using a mask, and a bottom indium (In) cathode was formed on the backside of the n-Si by welding.

### Fabrication of CNT/n-Si solar cells

The CNT/n-Si solar cells were fabricated following the procedure in the previous report^16^. CNT (1 mg, MEIJO eDIPS, EC grade; MEIJO Nanocarbon Co., Ltd., Nagoya, Japan) was dispersed in sodium dodecylbenzenesulfonate (SDBS, Sigma Aldrich, St. Louis, MO, USA) aqueous solution (0.5 wt%, 10 mL) by bath sonication for 3 min. The dispersion (100 μL) with water (~15 mL) was vacuum filtrated onto a hydrophilic membrane filter (pore size 0.1 μm, VCWP, Merck Millipore, Darmstadt, Germany) to form the CNT film. After filtration, the film was washed by filtrating purified water (100 mL), soaked in water to have it float on the water surface, and heated at 95 °C in a water bath for 70 min to remove the SDBS. The CNT film on the hot water was then scooped onto the n-Si substrate, and a drop of ethanol was placed and dried on the surface to build a good contact between CNT and n-Si. The top Au anode with 2.2 mm-sized opening was formed on the CNT film by sputtering using a mask, and the bottom In cathode was formed on the backside of the n-Si by welding.

### Characterization and measurement

The optical transmittance and sheet resistance measurements were performed by forming PEDOT:PSS and CNT films on quartz glass and polyethylene terephthalate (PET) substrates, respectively. Their optical transmittance was obtained by using ultraviolet-visible spectrophotometry (UV-Vis; V-630, JASCO, Tokyo, Japan) and the sheet resistance was measured by the four-point probe method. The passivation layers formed on the n-Si substrates were analyzed by X-ray photoelectron spectroscopy (XPS; JEOL JPS-9010TR, Tokyo, Japan) and spectroscopic ellipsometry (UVISEL ER AGMS iHR320, Tokyo, Japan). For the spectroscopic ellipsometry, three samples were prepared for each condition and three points were analyzed for each sample. The PV performance was evaluated by making four cells for each condition and analyzing them by a solar cell evaluation system (JASCO YQ-2000, Tokyo, Japan) under a solar simulator (CEP-2000MLQ, Bunko-keiki, Tokyo, Japan, xenon lamp, AM 1.5 G spectral illumination of 100 mW cm^−2^). The irradiation intensity was calibrated by a standard Si photodiode device (BS-500BK, SN/182). The external quantum efficiency (EQE) was evaluated for some cells by using a xenon lamp attached to a monochromator (300–1200 nm) by a CEP-2000 integrated system (Bunko-keiki, Tokyo, Japan). A light blocking mask with a hole (ϕ = 2 mm) made out of Ni foil with a size of 10 × 10 mm^2^ and a thickness of 50 μm was placed on the solar cell during evaluation to standardize the amount of incident light.

## Results and Discussion

### PEDOT:PSS/n-Si solar cells without and with passivation

**Figure 1:**
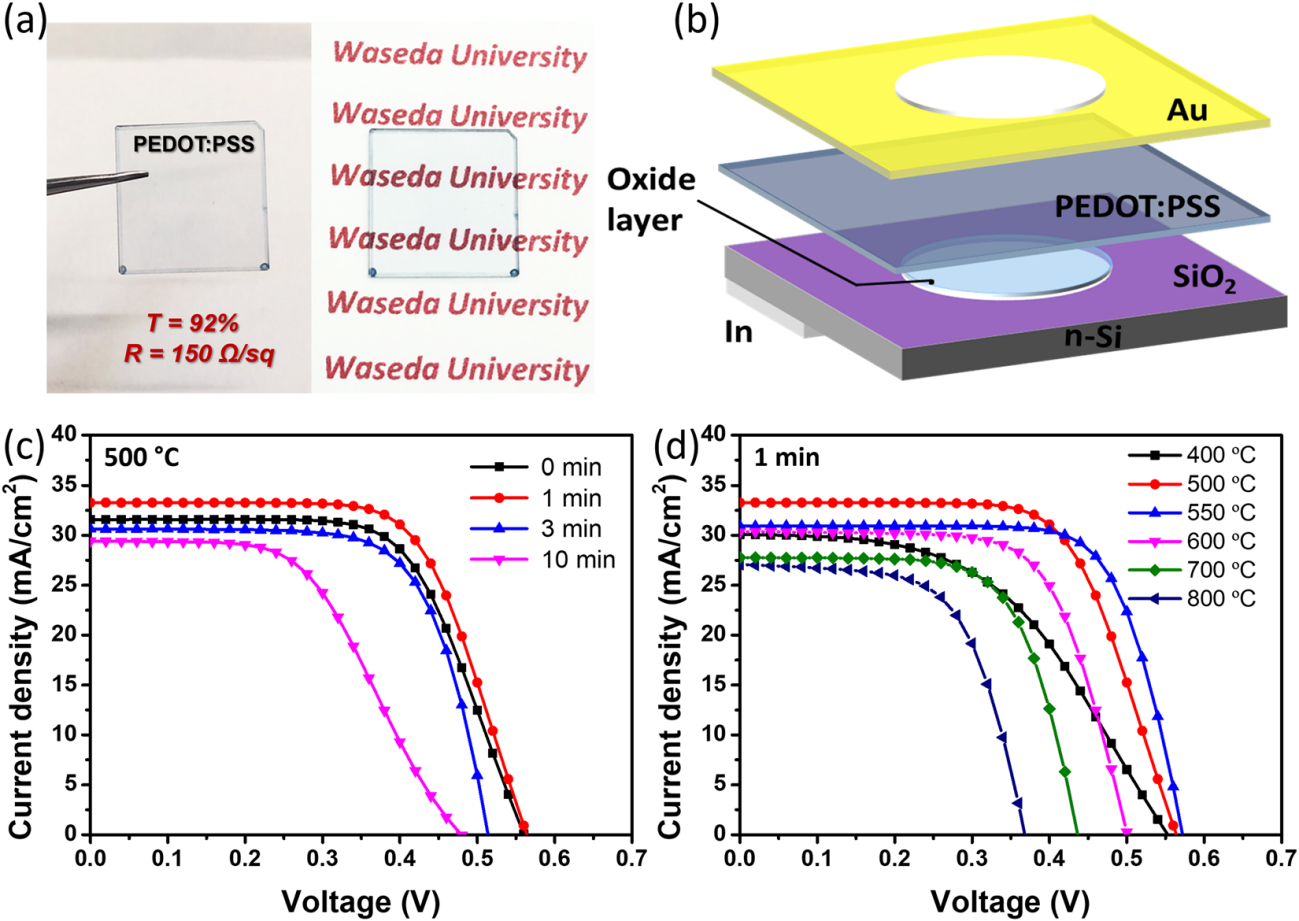
(**a**) The PEDOT:PSS film spin-coated on the quartz glass substrate. (**b**) A schematic of the PEDOT:PSS/n-Si heterojunction solar cell with the passivated oxide layer. The *J*–*V* curves of the PEDOT:PSS/n-Si heterojunction solar cells with n-Si substrates passivated (**c**) at 500 °C for 0–10 min and (**d**) at 400–800 °C for 1 min.

We first investigated the effect of the passivation on the PV performance for the PEDOT:PSS/n-Si heterojunction solar cells. PEDOT:PSS is one of the excellent and commonly used conductive materials due to the high conductivity, appropriate work function, and good film forming properties^25,26^. A PEDOT:PSS film was obtained on the passivated n-Si by spin-coating the PEDOT:PSS and IPA mixed solution. The thickness was controlled by modulating the spin-coating speed and time. The relationship between transmittance and resistance of the films are shown in Fig. S2. PEDOT:PSS films with an optical transmittance of *T* = 92% at 550 nm wavelength and a sheet resistance of 150 Ω/sq were used in this experiment (Fig. 1a). Figure 1b shows the schematic of the PEDOT:PSS/n-Si heterojunction solar cell with an oxide layer.

To investigate the passivation condition for the high PV performance of the PEDOT:PSS/n-Si heterojunction solar cells, the annealing time and temperature were changed over a wide range. Figure 1c shows the *J*–*V* curves of solar cells fabricated with n-Si passivated at 500 °C for 0, 1, 3, and 10 min. With increasing annealing time from 0, 1, to 3 min, FF increased from 0.65 to 0.71 while open circuit voltage (*V*_oc_) decreased from 0.56 to 0.52 V, leading to PCEs of 11.31%, 12.22%, and 10.96%. Further increase in annealing time resulted in poor FF. Thus we fixed the annealing time at 1 min. The *J*–*V* curves of solar cells fabricated with n-Si passivated at 400, 500, 550, 600, 700, and 800 °C for 1 min were shown in Fig. 1d. FF reached the highest value of 0.73 and yielded the high PCE of 12.65% by the passivation at 550 °C. As the temperature continued to increase, *V*_oc_ and short circuit current density (*J*_sc_) started to decrease which resulted in low PCEs. However, the FF was maintained at a relatively high value (above 0.60), indicating the formation of a high-quality passivation layer. The dark *J−V* characteristics are shown in Fig. S3 and the corresponding PV parameters of these solar cells are summarized in Table 1, revealing that the good passivation was achieved by quick annealing at 500–550 °C for 1 min.

**Table 1:** PV characteristics of the PEDOT:PSS/n-Si heterojunction solar cells with different passivation conditions.

Temperature (°C)	Time (min)	*J*_sc_ (mA/cm^2^)	*V*_oc_ (V)	FF	PCE (%)
500	0	31.26 ± 0.32	0.560 ± 0.002	0.652 ± 0.005	11.31 ± 0.15
500	1	32.64 ± 0.42	0.562 ± 0.008	0.676 ± 0.009	12.22 ± 0.23
500	3	30.41 ± 0.24	0.520 ± 0.007	0.707 ± 0.006	10.96 ± 0.10
500	10	29.67 ± 0.25	0.470 ± 0.010	0.513 ± 0.007	7.16 ± 0.18
400	1	29.90 ± 0.61	0.543 ± 0.006	0.503 ± 0.013	8.20 ± 0.04
550	1	30.84 ± 0.16	0.564 ± 0.005	0.725 ± 0.014	12.65 ± 0.18
600	1	30.13 ± 0.45	0.506 ± 0.011	0.675 ± 0.009	10.27 ± 0.12
700	1	27.62 ± 0.32	0.432 ± 0.003	0.674 ± 0.007	8.05 ± 0.08
800	1	27.07 ± 0.18	0.370 ± 0.005	0.625 ± 0.014	6.26 ± 0.17

Average and standard deviation are shown based on the results of the four cells fabricated for each condition.

**Figure 2:**
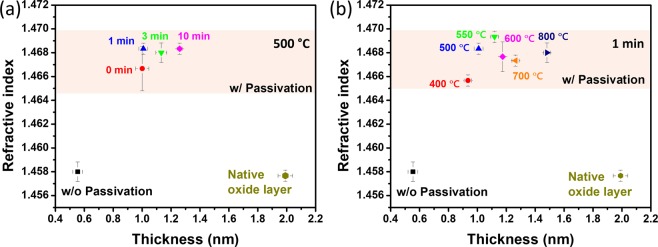
The spectroscopic ellipsometry analysis of the oxide layer on Si surface formed by annealing (**a**) at 500 °C for 0–10 min and (**b**) at 400–800 °C for 1 min. “w/o Passivation” represents the n-Si substrates after etching in HF, “native oxide” represents the n-Si substrates kept in air for 10 days after HF-treatment, and “w/ Passivation” represents the n-Si substrates with passivation by annealing after the HF-treatment. Three samples were prepared for each condition and three points were measured for each sample. Error bars show the standard deviation.

Then the n-Si substrates without and with passivation at 500 °C for 1 min were investigated by XPS (Fig. S4). The Si 2p spectra had a minor peak at ~102 eV, which can be assigned to SiO_*x*_ (Fig. S4b). Also the O 1 s spectra showed a peak located around 532.5 eV, which is consistent with the Si-O group (Fig. S4c). The intensities of the two peaks associated with SiO_*x*_ increased after annealing, showing the growth of SiO_*x*_ by annealing. Then the passivation layer prepared by various conditions were further characterized by ellipsometry. Three samples were prepared for each passivation condition and three points were analyzed for each sample (Fig. 2a,b). The n-Si substrate after etching in HF for 30 s had a surface layer with a thickness and refractive index of around 0.5–0.6 nm and 1.458, respectively. The n-Si substrates had surface layers with increased thicknesses of 0.9–1.5 nm, and the thickness increased as the annealing time and temperature increased. The refractive index was higher for the layer with annealing (1.465–1.470) than without annealing (~1.458), indicating a higher density for the oxide layer formed by annealing when compared to the native oxide layer on HF-treated Si surface. The low-pressure, quick annealing makes the oxygen feed small and the oxide layer thin while the high-temperature condition enhances the atomic diffusion and makes the structure of the oxide layer relax well.

Annealing at 500–550 °C for 1 min yielded good passivation layers of 1.0–1.1 nm (Fig. 2a,b) which significantly enhanced the PV performance (Fig. 1c,d). However, annealing at higher temperature or longer time made the oxide layer thicker, resulting in the degraded performance. Then we calculated the series resistance (*R*_s_) and shunt resistance (*R*_sh_) of the solar cells based on their *J*–*V* curves. *R*_s_ is related to the charge transport and the contacts between interfaces, and *R*_sh_ is a typical parameter that is related to manufacturing and internal defects^27,28^. This significant enhancement in FF was revealed by the lower *R*_s_ and higher *R*_sh_ of the solar cells (Table 1, Fig. S5). From the *R*_s_ and *R*_sh_ of the devices, we deduced the working mechanism of the passivation layer. When the oxide layer has insufficient thickness and/or density, the carrier recombination is not suppressed efficiently, resulting in a large dark current similar with the solar cells without passivation^29^. With the proper annealing, the dense and thin oxide layer forms on the n-Si surface, which passivates the dangling bonds on the n-Si surface to enhance *R*_sh_ and diode properties while permitting the hole transport by the tunneling effect. Further thickening of the oxide layer impedes the hole transport via the tunneling effect, leading to a significantly increased *R*_s_. The dense and thin oxide layer works as a high-quality hole transport layer and passivation layer for the solar cells, resulting in the enhancements of FF and PCE.

**Figure 3:**
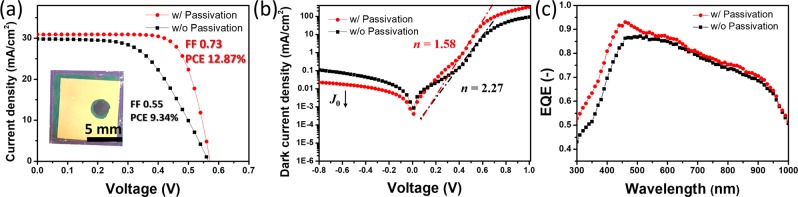
(**a**) *J*–*V* curves for the PEDOT:PSS/n-Si heterojunction solar cells without (w/o) and with (w) passivation of the Si surface. The inset of (**a**) is the digital image of the solar cells with passivation. (**b**) Log *J*–*V* curves of the champion PEDOT:PSS/n-Si heterojunction solar cells in the dark. (**c**) The EQE spectra of the champion PEDOT:PSS/n-Si solar cells without and with passivation under AM 1.5 G irradiation.

After annealing at 550 °C for 1 min, the PEDOT:PSS/n-Si solar cell showed superior PV characteristics (Figs. 3a and S6), with a *J*_sc_ of 30.94 mA/cm^2^, an *V*_oc_ of 0.57 V, and a FF of 0.73, yielding the best PCE of 12.87%, which is 1.38-times higher than that of the device without passivation (9.34%). Moreover, to further investigate the diode properties, the dark *J−V* characteristics of solar cells were measured. The PEDOT:PSS/n-Si solar cells with passivation exhibited better diode properties than its counterpart without passivation. Figure 3b shows lower reverse saturation current density (*J*_0_) and diode ideality factor (*n*) for the PEDOT:PSS/n-Si solar cells with passivation in the dark, which indicates lower carrier recombination with the improved passivation quality of the oxide layer. The EQEs of the solar cells are shown in Fig. 3c, revealing the incident photon-to-current efficiency in the cells. The EQE of the PEDOT:PSS/n-Si solar cell with passivation reached 0.93 at maximum, which is slightly larger than that of the cells without passivation in the wavelength range between 400 and 900 nm. Fig. S7 shows the calculated integrated current density by using the overlap between the EQE spectrum and the AM 1.5 G solar photon flux. The calculated integrated photocurrent yields current densities of 30.28 and 28.65 mA/cm^2^ for the PEDOT:PSS/n-Si solar cells with and without passivation, which are consistent with the photocurrent densities determined from the *J–V* curves. It should be noted that the improved PV performance was achieved without any chemical doping or anti-reflective layers.

### CNT/n-Si solar cells without and with passivation

**Figure 4:**
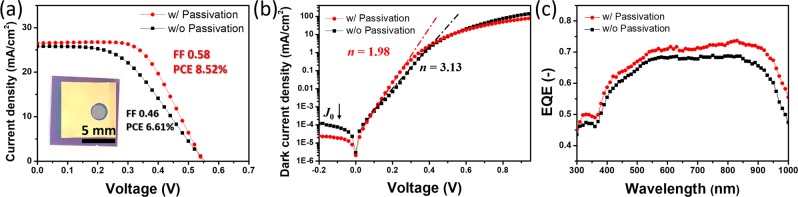
(**a**) *J*–*V* curves of the champion CNT/n-Si heterojunction solar cells without and with passivation. The inset of (**a**) is the digital image of the solar cell with passivation. (**b**) Log *J*–*V* curves of the champion CNT/n-Si heterojunction solar cells in the dark. (**c**) The EQE spectra of the champion CNT/n-Si heterojunction solar cells without and with passivation under AM 1.5 G irradiation.

To determine if a similar improvement in PV performance could be achieved for other n-Si heterojunction solar cells, CNT/n-Si heterojunction solar cells were fabricated based on the passivated n-Si substrates and CNT films. In our previous study, we developed a repetitive dispersion-extraction process which transforms CNT powder to high-quality CNT films at high yields without significant damage to CNT^30^. The optical transmittance of CNT films was controlled by changing the quantity of CNT solution (Fig. S8). In this study, CNT films with an optical transmittance of *T* = 90% and a sheet resistance of 208 Ω/sq without doping were used in making the CNT/n-Si cells (Fig. S9). After passivating the n-Si substrate by quick annealing in vacuum at 550 °C for 1 min, a CNT film was transferred to the passivated n-Si substrate to form the heterojunction junction device. Fig. S9b shows a schematic of the CNT/n-Si heterojunction solar cell with the oxide layer. The *J*–*V* curves of the cells with and without passivation are shown in Fig. 4a with the inset photo of the cell with passivation. Apparently, CNT/n-Si solar cells with passivation (see also Fig. S10) exhibit better PV performance, with a *J*_sc_, *V*_oc_, and FF of 26.53 mA/cm^2^, 0.55 V, 0.58, respectively, yielding the PCE of 8.52%, which is 1.29-times higher than that of the device without passivation (6.61%). Figure 4b shows the dark *J*–*V* characteristics of the solar cells; the lowered *J*_0_ and *n* indicate that the interface carrier recombination is suppressed after the passivation. The EQE was enhanced over a broad wavelength range from 400 to 1000 nm (Fig. 4c) and the calculated integrated current density showed a slightly higher value (26.20 mA/cm^2^) compared with the solar cells without passivation (24.71 mA/cm^2^) (Fig. S11), which are consistent with the *J*–*V* characteristics. These results indicate that, similarly to the case of the PEDOT:PSS/n-Si solar cells, the oxide layer plays an essential role by acting as the hole transport layer and the passivation layer, leading to a considerable improvement in the PV performance of the CNT/n-Si heterojunction solar cells.

### Stability of the PEDOT:PSS/n-Si and CNT/n-Si heterojunction solar cells

**Figure 5:**
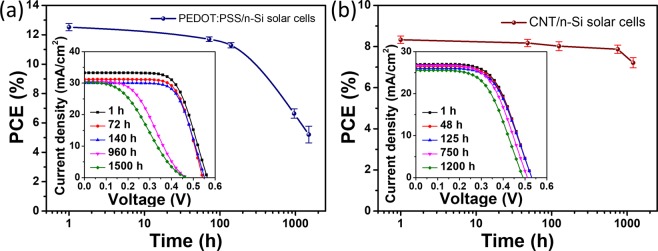
PCEs of (**a**) the PEDOT:PSS/n-Si heterojunction solar cells and (**b**) the CNT/n-Si heterojunction solar cells as a function of storage time in the air without illumination and encapsulation. n-Si substrates were passivated by annealing at 550 °C for 1 min. Three to five cells were fabricated and evaluated for each type, and the error bars show the standard deviation. The insets show the time evolution of the *J*–*V* curves of a typical cell for each type.

Thus far, we fabricated the PEDOT:PSS/n-Si and CNT/n-Si heterojunction solar cells based on the n-Si substrates passivated by a quick annealing process, which effectively enhanced the PV performances of the devices. We then made a preliminary study on the stability of these hybrid heterojunction solar cells by storing them in the air at room temperature without illumination and encapsulation. Figure 5 shows the measured efficiency evolution of the PEDOT:PSS/n-Si and CNT/n-Si cells with initial PCEs of 12.64% and 8.52%, respectively. A noticeable degradation in PCE was observed for the PEDOT:PSS/n-Si solar cell after ~1000 h; the degradation is possibly related to the interaction of PEDOT:PSS/n-Si junction with the moisture in the air^31^. PEDOT:PSS would absorb the moisture and form interface dipoles, resulting in S-shaped *J*–*V* curves (960 and 1500 h). *R*_s_ increases drastically from 2.21 to 10.27 Ω cm^2^, and the FF decreases from 0.73 to 0.37, resulting in the low PCE of 5.21% after 1500 h. However, the CNT/n-Si solar cell exhibited better durability than the PEDOT:PSS/n-Si solar cell due to the better stability of CNT. The PCE decreased to 7.01% after storing for 1200 h in air. It is expected that the chemical doping or encapsulation layer will further improve the stability of these solar cells^32^. Effective doping of the heterojunctions is now under study.

To achieve very low-cost solar cells, breakthrough is needed not only for the formation of the junctions but also for the production of crystalline Si substrates. We have previously developed a “rapid vapor deposition of liquid Si and *in situ* melt crystallization” method that enables deposition of lager-grain (>100 μm) continuous polycrystalline Si films in 1 min^33,34^. The resulting Si films are quickly cooled down from ~1400 °C in vacuum, thus the *in situ* surface passivation can be expected. The quick processes of the Si film fabrication, the Si surface passivation, and the printable heterojunction will be combined toward very low-cost, heterojunction solar cells.

## Conclusions

We report a simple and efficient passivation method for n-Si substrates. A thin and dense oxide layer was grown on the Si surface by a quick annealing process in vacuum (1 min, 500–550 °C, base and working pressures of <5 × 10^−4^ and < 2 × 10^−2^ Pa), which greatly cut down the passivation time and cost. With the passivation layer, the PV performances of the PEDOT:PSS/n-Si and CNT/n-Si heterojunction solar cells were effectively enhanced. The best performing PEDOT:PSS/n-Si and CNT/n-Si solar cells achieved high PCEs of 12.87% and 8.52%, with the remarkably enhanced FFs of 0.73 and 0.58, respectively. The presence of the dense oxide layer with the thickness around 1.1 nm greatly suppressed the carrier recombination while allowing the hole transport by tunneling effect. The simple surface passivation enhanced the PV performance of the Si-based heterojunction cells with various materials without losing the easiness of the cell fabrication.

## Supplementary information

Supplementary Information

## Data Availability

All data generated or analysed during this study are included in this published article and its Supplementary Information file.
