# Supplementary material for: Enhancing the photovoltaic performance of hybrid heterojunction solar cells by passivation of silicon surface via a simple 1-min annealing process
Source: Sci Rep. 2019 Aug 19;9:12051. doi: 10.1038/s41598-019-48504-7 (PMC6700085; doi:10.1038/s41598-019-48504-7)
Supplement: Supplementary file 1 — Supplementary Information [file 41598_2019_48504_MOESM1_ESM.pdf]

Supplementary information

Enhancing the photovoltaic performance of hybrid heterojunction solar cells by passivation of silicon surface via a simple 1-min annealing process

Rongbin Xie[1], **Naoya Ishijima**[1], **Hisashi Sugime**[1], **and Suguru Noda**[1,2,*]

[1] Department of Applied Chemistry, Waseda University, Tokyo 169-8555, Japan
[2] Waseda Research Institute for Science and Technology, Waseda University, Tokyo 169-8555, Japan
[*] noda@waseda.jp

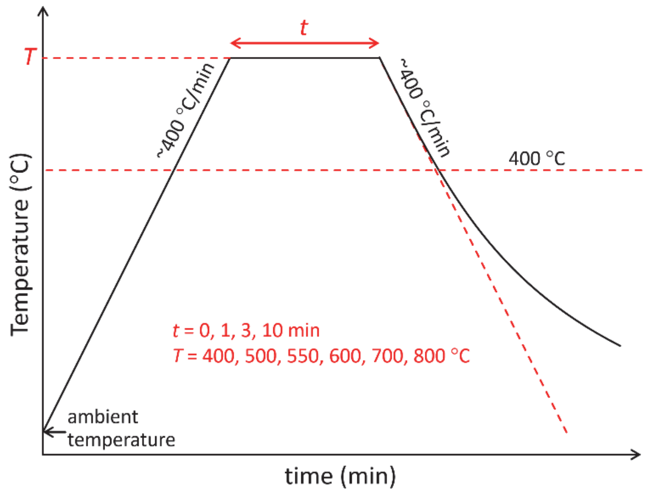

Figure S1. A process flow showing the substrate temperature with time during the annealing process.

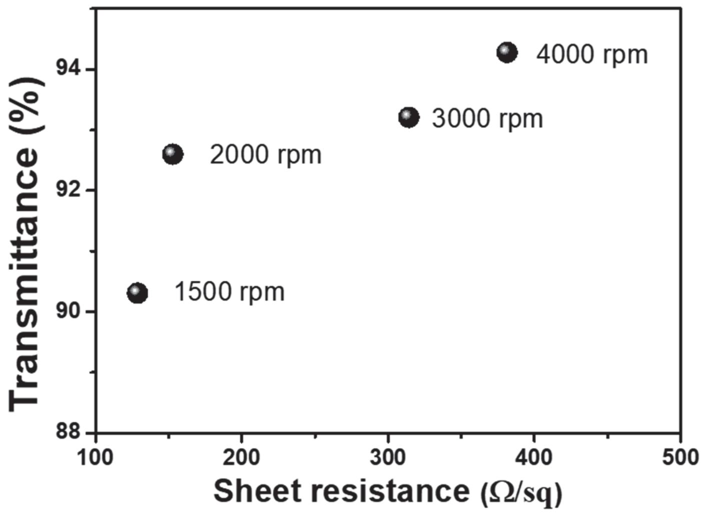

Figure S2. The relationship between the optical transmittance at 550 nm and sheet resistance of the PEDOT:PSS films spin-coated on quartz glass substrates.

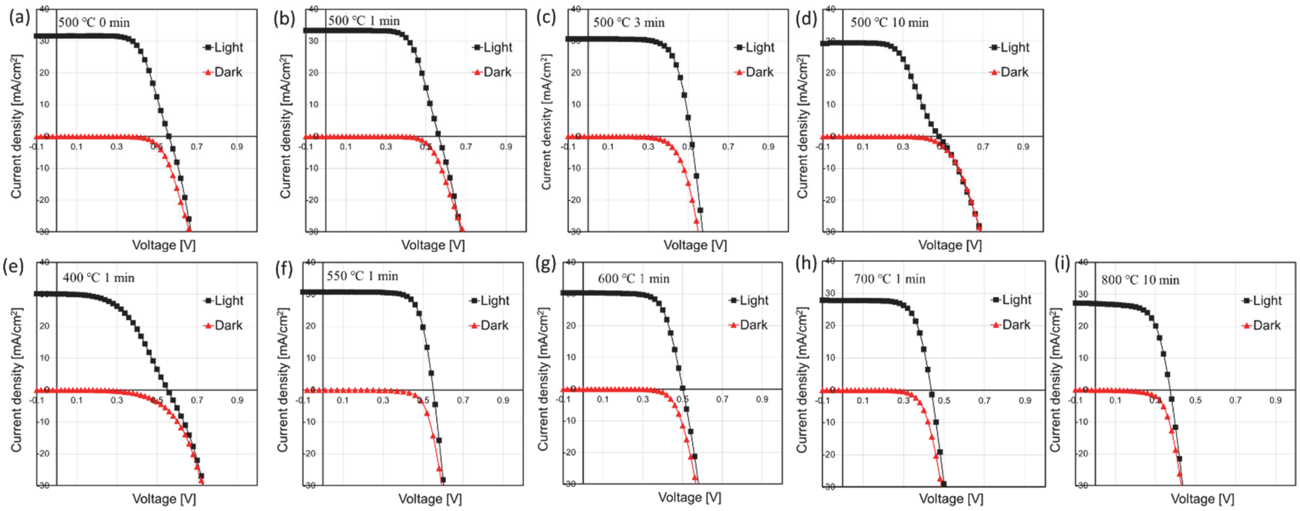

Figure S3. The *J-V* curves of PEDOT:PSS/n-Si solar cells fabricated with the n-Si substrates with different passivation conditions; 500 °C for (a) 0 min, (b) 1 min, (c) 3 min, (d) 10 min and 1 min at (e) 400 °C, (f) 550 °C, (g) 600 °C, (h) 700 °C, (i) 800 °C.

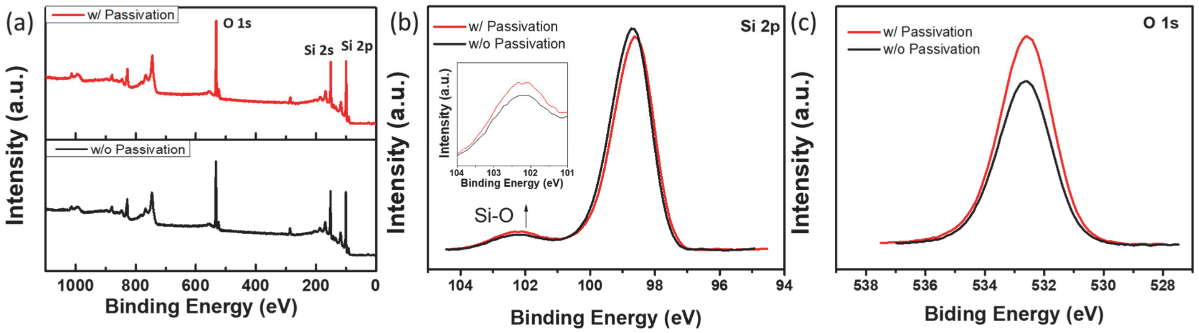

Figure S4. XPS spectra of n-Si substrates without and with passivation by annealing at 500 °C for 1 min. (a) Survey spectra. (b) Si 2p spectra. (c) O 1s spectra.

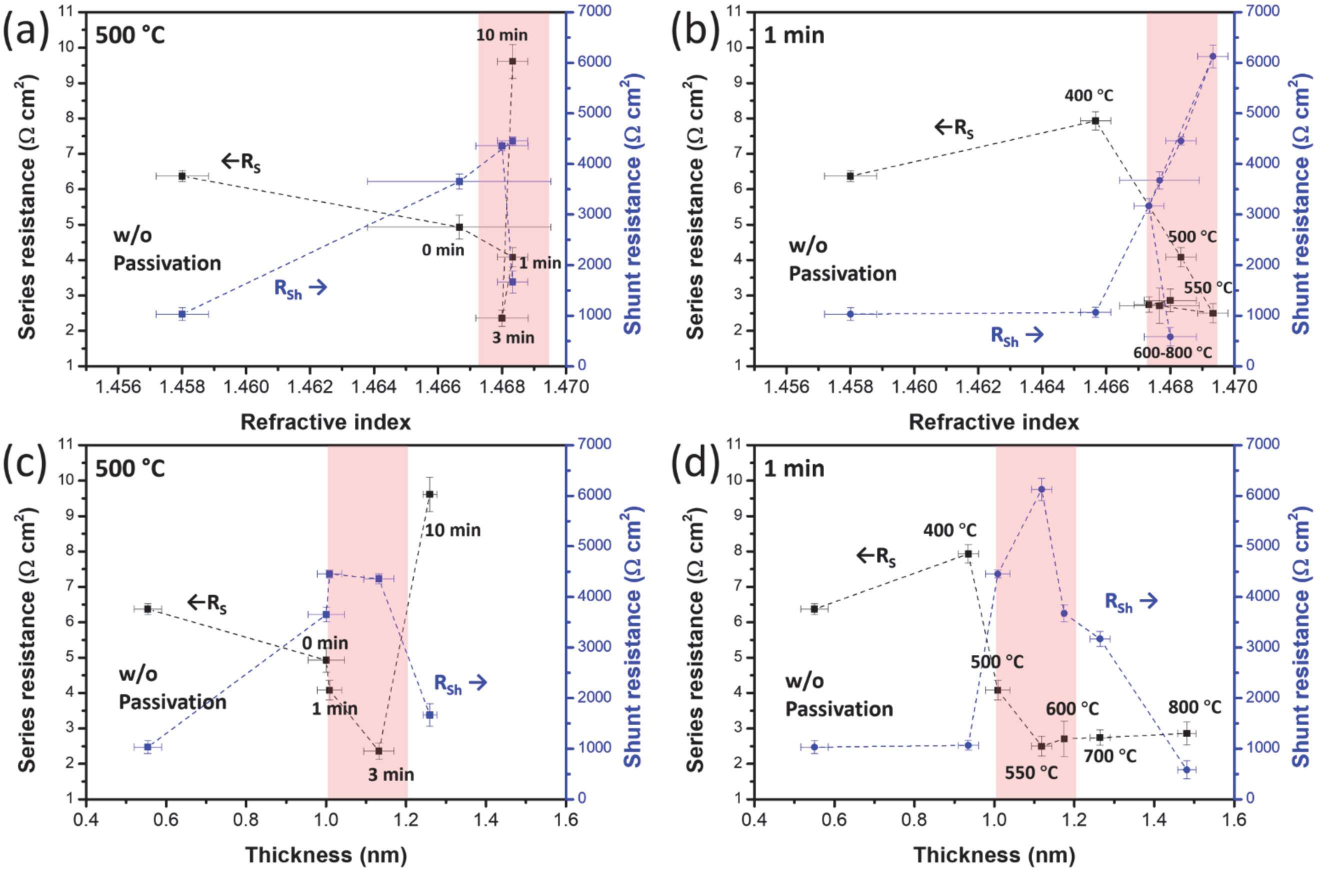

Figure S5. Comparison of R_s and R_{sh} of the PEDOT:PSS/n-Si solar cells with oxide layer of different refractive indices (a,b) and thicknesses (c,d). The oxide layers were grown (a,c) at 500 °C for 0–10 min and (b,d) at 400–800 °C for 1 min. The dash-dotted lines are drawn to guide the eye. R_s and R_{sh} are the approximate values calculated from the slope of the *J-V* curve.

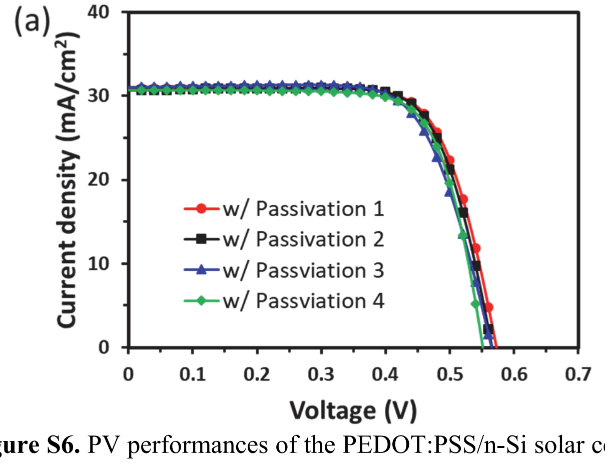

(b)

	J_{sc} [mA/cm²]	V_{oc} [V]	FF	PCE [%]
w/ Passivation 1	30.94	0.57	0.73	12.87
w/ Passivation 2	30.69	0.57	0.73	12.79
w/ Passivation 3	31.05	0.57	0.70	12.44
w/ Passivation 4	30.69	0.56	0.74	12.50

Figure S6. PV performances of the PEDOT:PSS/n-Si solar cells fabricated with the optimum passivation condition (1 min, 550 °C). (a) *J-V* curves. (b) PV parameters.

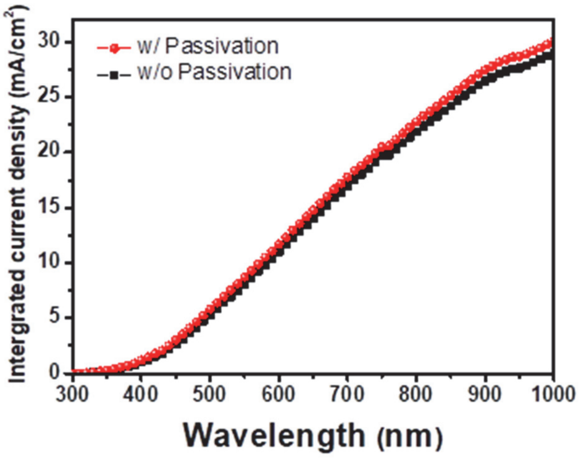

Figure S7. The integrated photocurrent measured for the PEDOT:PSS/n-Si solar cells without and with passivation under AM 1.5G irradiation.

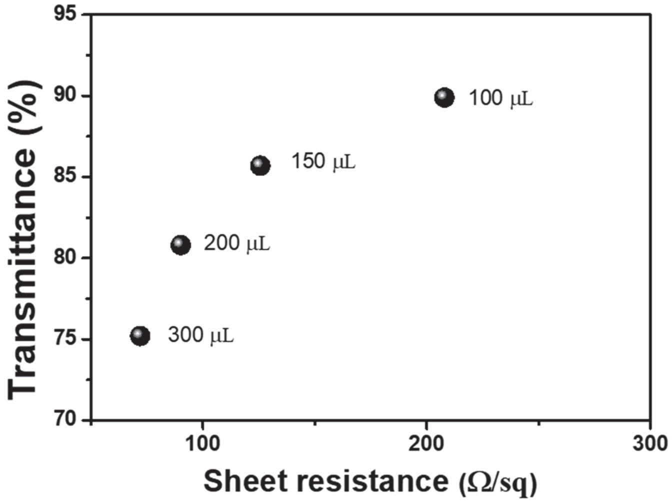

Figure S8. The relationship between the optical transmittance at 550 nm and sheet resistance of the CNT films transferred to PET substrates.

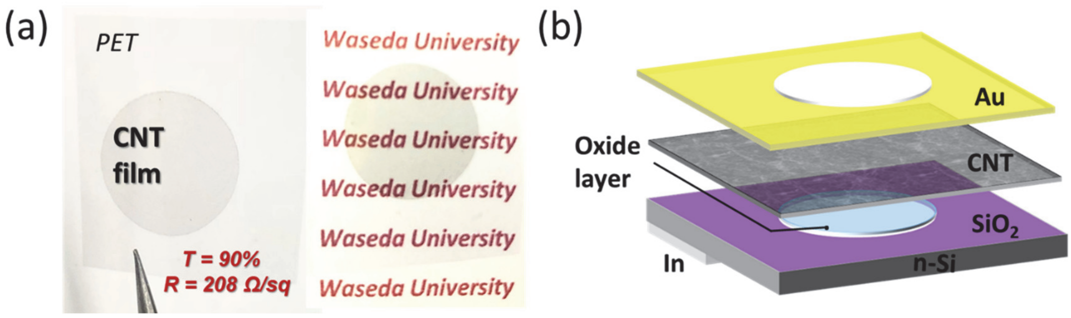

Figure S9. (a) Optical image of a transparent CNT film on a PET film substrate. (b) A schematic of the CNT/n-Si heterojunction solar cell with the passivated oxide layer.

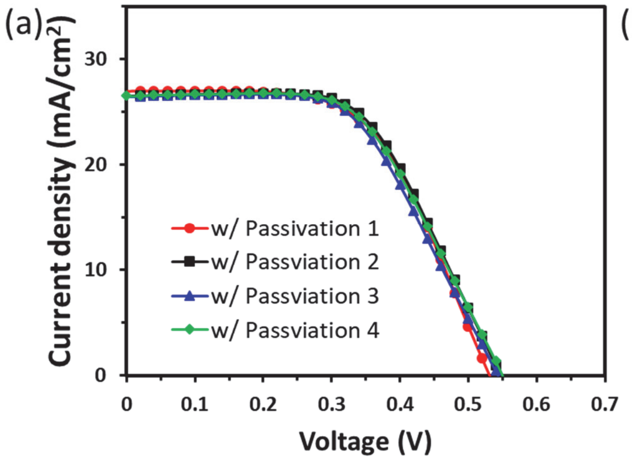

(b)

	J_{sc} [mA/cm²]	V_{oc} [V]	FF	PCE [%]
**w/ Passivation 1**	26.94	0.53	0.57	8.33
**w/ Passivation 2**	26.53	0.55	0.58	8.52
**w/ Passivation 3**	26.49	0.55	0.56	8.15
**w/ Passivation 4**	26.54	0.55	0.56	8.34

Figure S10. PV performances of the CNT/n-Si solar cells fabricated with the optimum passivation condition (1 min, 550 °C). (a) *J-V* curves. (b) PV parameters.

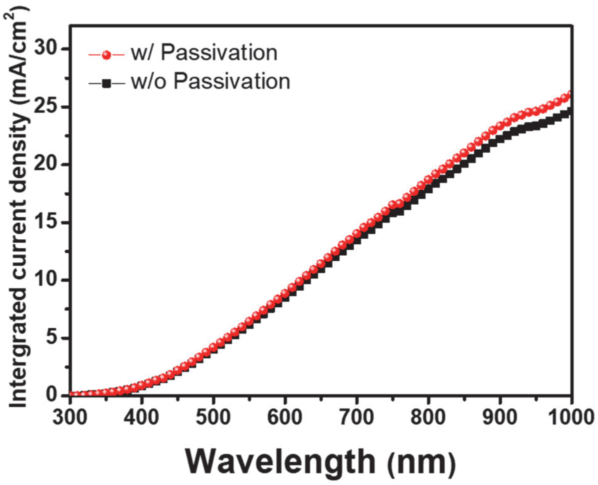

Figure S11. The integrated photocurrent the CNT/n-Si solar cells without and with passivation under AM 1.5G irradiation.
